# Supplementary material for: Digitalization of adverse event management in oncology to improve treatment outcome—A prospective study protocol
Source: PLoS One. 2021 Jun 4;16(6):e0252493. doi: 10.1371/journal.pone.0252493 (PMC8177479; doi:10.1371/journal.pone.0252493)
Supplement: S1 File — (DOCX) [file pone.0252493.s001.docx]

| Section/item | ItemNo | Description |
| --- | --- | --- |
| **Administrative information** | | |
| Title | 1 | NEMO - an app for side effect management in oncology |
| Trial registration | 2a | NCT04493450 |
| Protocol version | 3 | Version 1.3 / 14.09.2020 |
| Funding | 4 | This study receives no funding |
| Roles and responsibilities | 5a | Prof. Dr. Thomas Seufferlein, Clinic for Internal Medicine I, Ulm (Study leader, Protocol Committee)  Prof. Dr. Hans A. Kestler, Institute of Medical Systems Biology, Ulm (Study leader, Protocol Committee)  Dr. Angelika Kestler, Clinic for Internal Medicine I, Ulm (Study coordinator, Protocol Committee)  Dr. Peter Kuhn, Comprehensive cancer centere Ulm (trusted third party)  Dr. Johann M. Kraus, Institute of Medical Systems Biology, Ulm (Statistic and biometry)  M.sc. Silke D. Kühlwein, Institute of Medical System Biology (Protocol Commiteee)  Dr. Julian D. Schwab, Institute of Medical System Biology (Protocol Commiteee)  M.sc. Robin Szekely, Institute of Medical System Biology (Protocol Commiteee)  M.sc. Patrick Thiam, Institute of Medical System Biology (Protocol Commiteee)  Dr. Rolf Hühne, Institute of Medical System Biology (Protocol Commiteee)  Dr. Axel Fürstberger, Institute of Medical System Biology (Protocol Commiteee)  Franz Jobst, Ulm University Clinic (Protocol Commiteee) |
|  | 5b | Prof. Dr. Thomas Seufferlein, Clinic for Internal Medicine I, University Hospital Ulm |
|  | 5c | Prof. Dr. Thomas Seufferlein is study leader and coordinator of the clinical trial. He was involved in the conceptualisation of the study. |
|  | 5d | Prof. Dr. Thomas Seufferlein, Clinic for Internal Medicine I, Ulm (Study leader, Protocol Committee)  Prof. Dr. Hans A. Kestler, Institute of Medical Systems Biology, Ulm (Study leader, Protocol Committee)  Dr. med. Angelika Kestler, Clinic for Internal Medicine I, Ulm (Study coordinator, Protocol Committee)  Dr. Peter Kuhn, Comprehensive cancer centere Ulm (trusted third party)  Dr. Johann M. Kraus, Institute of Medical Systems Biology, Ulm (Statistic and biometry)  M.sc. Silke D. Kühlwein, Institute of Medical System Biology (Protocol Commiteee)  Dr. Julian D. Schwab, Institute of Medical System Biology (Protocol Commiteee)  M.sc. Robin Szekely, Institute of Medical System Biology (Protocol Commiteee)  M.sc. Patrick Thiam, Institute of Medical System Biology (Protocol Commiteee)  Dr. Rolf Hühne, Institute of Medical System Biology (Protocol Commiteee)  Dr. Axel Fürstberger, Institute of Medical System Biology (Protocol Commiteee)  Franz Jobst, Ulm University Clinic (Protocol Commiteee) |
| Introduction |  |  |
| Background and rationale | 6a | Due to the respective tumor therapy protocols, but also due to limited resources, doctors only see their outpatients at certain intervals. Patients often forget important information about side effects, intolerances and general well-being or do not consider this information relevant.  In particular, drug combination therapies in oncology often contain active ingredients that can cause side effects such as nausea, inflammation of the mucous membrane (mucositis), diarrhea (diarrhea) or polyneuropathy. The acceptance of tumor therapy is decisively determined by the occurrence and duration of the side effects. If the therapy is well tolerated by the reduction of side effects, an improvement in quality of life is achieved. This is an important goal of a tumor therapy that also takes into account the psycho-oncological and social needs of the patients.  For the standardized documentation of possible relevant side effects and other therapy-associated information, an app has been developed to make it easier for patients to record these side effects.  In a first part, the acceptance and applicability of the app will be tested within the scope of the present study. If this first part is completed with positive results, a second phase is planned to investigate the effect of using the app on the management of side effects and their supportive therapy, as well as the maintenance of the original chemotherapy dose. |
|  | 6b | We examine the impact of new documentation media. Therefore, we compare a group of patients who report the occurred adverse events via the NEMO smartphone app and patients using standard reporting procedure. |
| Objectives | 7 | Patient acceptance of a new means of communication, Reduction of side effects by at least 1 degree according to CTCAE at study start. |
| Trial design | 8 | This study will be implemented in two parts. The first part should evaluate the feasibility and acceptance of a smartphone-based adverse reaction documentation. It will be followed by a second part. which will compare the impact of smartphone reported adverse events regarding applied therapy doses to those of patient with standard care. For the detection of occurred adverse events at an early stage, the participants will answer a standardized questionnaire (Table 1 and 2) via the NEMO app every day. Depending on the underlying therapy scheme, the NEMO questionnaire can be selected for chemotherapy (Table 1) or combined immunotherapy (Table 2), targeted therapy, and chemotherapy. NEMO allows for switching the therapeutic settings in case the therapeutic scheme changes. During therapy sessions, the documented data is transmitted securely via QR-codes to the treating physician and used for future therapy settings. To assess the. feasibility, 30 patients will be divided non-randomly into three age groups (< 55 years, 55-75 years, >75 years) of 10 patients each. These people will be followed over six months. Participants will answer the respective NEMO questionnaire about adverse events on a daily basis over these six months. After six months, they will answer a questionnaire about user-friendliness and feasibility (Table 3). Evaluation of these questionnaires allows for studying the different patients' characters and their potential benefit of using NEMO. The answered questionnaires could also give insights to identify barriers to answering the questions. The results of this evaluation enable us to amend the criteria for recruiting patients of the second part, such as their age and improving the application for the second part of the study. To assess the reduction of adverse events, 36 participants will be randomly assigned a group of 18 persons (smartphone user and standard care). Again, smartphone users will answer their respective NEMO questionnaires every day about occurred adverse events for six months. The transfer of their reported data takes place every second month in parallel with the reevaluation of tumor therapy. Participants of the standard care group will report their occurred adverse events and duration only during their personal appointments. What the patient remembers and reports depends on each participant and is very individual. Based on these reported adverse events, physicians will delineate a CTCAE scoring. Physicians will additionally fill out an electronic case report form containing tumor stage and information about therapeutic substances and doses. Moreover, patients of both study arms will answer a questionnaire about life quality which is based on the European Organization for Research and Treatment of Cancer (EORTC) questionnaire at enrolment and once at the end of the study to investigate whether NEMO can improve the quality of life. |
| Methods: Participants, interventions, and outcomes | | |
| Study setting | 9 | Study will be conducted at the University Hospital Ulm |
| Eligibility criteria | 10 | Inclusion Criteria:   - infusional combination therapy with at least two chemotherapy substances with or without further targeted therapies - combination of at least two immune-checkpoint inhibitors - able to read and understand German   Exclusion Criteria:  Participants who meet any of the following criteria will be excluded:   - severe neurological disorders   severe psychiatric disorders |
| Interventions | 11a | Not applicable. The study is to analyse the potential of new media for documentation. This can be considered as observational study. |
|  | 11b | Criteria for discontinuing are death, leaving Ulm or acquiring of exclusion criteria. |
|  | 11c | NEMO provides a user friendly interface for elderly individuals (clear design, big fonts, and auditory feedback) helping an easy fill in of questionnaires. |
|  | 11d | There are no interventions that are permitted or prohibited during the trial. |
| Outcomes | 12 | Primary Outcome:  Patient acceptance of a new medium of communication  Change of occurred adverse events  Secondary Outcome:  Maintenance of chemotherapy doses through better adverse event management  Change in the quality of life |
| Participant timeline | 13 | Both parts of the study are planned to last 6 months and build on each other (see Fig 1 and Table 4). |
| Sample size | 14 | Sample size was determined with the statistical software G*Power (v3.1.9.4) We assumed an effect size of 1, corresponding to a side effect variation of 1 on the Likert scale, a type I error rate = 0.05, and a power of 80%. Planning for a Mann-Whitney test comparing the difference between two groups led to a required sample size of 18 individuals per group (Fig 2). |
| Recruitment | 15 | The first part will be used to assed the right group of subjected that later on will allow random assignment in the second part of the study. |
| **Methods: Assignment of interventions (for controlled trials)** | | |
| Allocation: |  |  |
| Sequence generation | 16a | The physician's desktop application belonging to the app provides the pseudonym NEMO-Identifier for the participating test person. This is an isolated system that is independent of the smartphone app. |
| Allocation concealment mechanism | 16b | Method of random allocation of patient will be set in at the time of the beginning of the study. |
| Implementation | 16c | The study coordinator will take care of assigning patients to random allocation into the two groups. |
| Blinding (masking) | 17a | Patients and physicians will be aware of the allocation. Given the rational of the study, no blinding is required. Committee members in charge of data analysis will only receive pseudonymised patient data. |
|  | 17b | No blinding is required. |
| **Methods: Data collection, management, and analysis** | | |
| Data collection methods | 18a | For the daily recording of occurring side effects, participants fill out a questionnaire on their smartphones (Tables 1 and 2). This collected data can be transferred to the desktop application of the treating physician by means of QR code, where a visual representation of the logged side effects is displayed.  No personal data is stored in the app itself. Here, data on pain, food intake and well-being are queried and stored with the respective CTCAE score.  The corresponding desktop application of the physician records the master data (surname, first name and date of birth) of the test person as well as the side effect scores transmitted by the test person.  For the evaluation of the app or the quality of life, additional paper questionnaires (EORTC-QLQ-C30) are answered by the participants in handwriting with ballpoint pens. Clinical data are documented by the treating physician using data sheets of the electronic Case Report Form. |
|  | 18b | We set up in NEMO reminders for the patients in order to minimize potential missing data points and increase compliance. |
| Data management | 19 | smartphone app runs exclusively offline and consequently avoids transmission over potentially insecure internet connections to store data on remote servers. Information is passed to physicians via QR code systems. |
| Statistical methods | 20a | Wilcoxon-Mann-Whitney test will be used to assess known-groups validity, as well as to compare differences between NEMO users and patients with standard reporting procedure concerning the severity of occurred adverse events. NEMO uses a Likert scale to score each questionnaire item. To rate the overall effect of adverse event management, a summary score will be calculated from a weighted sum of questionnaire items by the maximum number of items. Spearman correlation will be used to evaluate convergent validity between each item and the summary score of the quality of life questionnaire or its symptom scales. The impact of the side effect management will be analyzed by comparing changes in an item's scoring between the reevaluation steps during tumor therapy. This comparison will be made using a Jonckheere-Terpstra test |
|  | 20b | All statistical details have been given. |
|  | 20c | We set up in NEMO reminders for the patients in order to minimize potential missing data points. |
| **Methods: Monitoring** | | |
| Data monitoring | 21a | Members of the protocol committee and biometry and statistical analysis will be in charge of monitoring the pseudonymised data. Statical approach for analysis is described above in section 20a-c. |
|  | 21b | For exclusion we refer to 10c. Interim data will be accessed by the committee described in 21a. Study leaders and coordinator will be in charge of deciding for study termination. |
| Harms | 22 | Not applicable to the trial. The aim of the trial is to assess new media communication for report and reduction of adverse events. |
| Auditing | 23 | Not applicable to the trial. |
| Ethics and dissemination | | |
| Research ethics approval | 24 | Consent was obtained in a written form by the ethics committee Ulm University, Ulm, Germany - Proposal no. 406/19. |
| Protocol amendments | 25 | All relevant involved parties will be informed of important protocol modifications. |
| Consent or assent | 26a | All patients will have to sign a written declaration of consent in order to participate to the study. All participants will be informed of procedure and endpoint. They will be also given the possibility to ask questions to coordinators before consenting to take part to the study. Also, possibility of withdrawing consent at any time is an option. |
|  | 26b | This point is not applicable to the study. No biological specimens will be taken. |
| Confidentiality | 27 | Patients data will be passed to clinicians in offline modality and through QR code. |
| Declaration of interests | 28 | No competing interests for principal investigators is declared. |
| Access to data | 29 | In the study phase, the desktop application, which stores all master data of the test persons on a computer that is protected against access, is the data storage. In a later phase, a login system for the desktop application will be set up. It is planned to integrate the application into the hospital's clinical information system to ensure legally compliant documentation and data protection of personal data according to the applicable standards. Technical staff of the project will only receive data in pseudonymized form. Thus, it is not possible for these employees to draw conclusions about individual persons.  For the evaluation of the study, the data is transferred to the Trusted Third Party, pseudonymized and released for analysis. Additionally, the Trusted Third Party checks the plausibility of the data (monitoring). |
| Ancillary and post-trial care | 30 | The study has an observatory nature. No drugs or treatments will be provided, and therefore potential harm to participants is not possible. |
| Dissemination policy | 31a | After evaluation of the data, a prompt and speedy publication of the results is planned. There will be a publication to answer the main question (regardless of the results). |
|  | 31b | As authors are involved: The study committee as well as the principal investigators of the study center, who contributed significantly to the recruitment by including more than 10% of the total number of study participants. Additional authors will be included for evaluation or manuscript preparation according to their scientific input. |
|  | 31c | Study protocol is planned to be published under open access. |
| Appendices |  |  |
| Informed consent materials | 32 | Figure 1: Trial design  Figure 2: Sample size estimation  Table 1: Smartphone questionnaire chemotherapy  Table 2: Smartphone questionnaire immunotherapy  Table 3: Feasibility questionnaire  Table 4: Flow chart of study procedure |
| Biological specimens | 33 | No biological specimens will be used in the trial. |

Figure 1: Trial design

Figure 2: Sample size estimation

Table 1: Smartphone questionnaire chemotherapy


Table 2: Smartphone questionnaire immunotherapy

Table 3: Feasibility questionnaire

Table 4: Flow chart of study procedure
