# Supplementary material for: Digitalization of adverse event management in oncology to improve treatment outcome—A prospective study protocol
Source: PLoS One. 2021 Jun 4;16(6):e0252493. doi: 10.1371/journal.pone.0252493 (PMC8177479; doi:10.1371/journal.pone.0252493)
Supplement: S2 File — (DOCX) [file pone.0252493.s002.docx]

**NEMO – eine App für das Nebenwirkungs-Management in der Onkologie**

Trial/NCT-No.: **NCT04493450** Protokoll-Code: **NEMO**

Version/Datum: **Amendement 1.3 / 14.09.2020**

# 1. Allgemeine Informationen

## 1.1 Studienleitung

| **Studienleitung** |
| --- |
| **Prof. Dr. med. Thomas Seufferlein**  Klinik für Innere Medizin I, Universitätsklinikum Ulm  **Prof. Dr. rer. nat. Hans A. Kestler**  Institut für Medizinische Systembiologie, Universität Ulm |
| **Studienkoordination** |
| **Dr. med. Angelika Kestler**  Klinik für Innere Medizin I, Universitätsklinikum Ulm |
| **Vertrauenswürdige dritte Partei/Datentreuhändler (Trusted Third Party)** |
| Dr. hum. biol. Peter Kuhn  Comprehensive Cancer Center Ulm, Universitätsklinikum Ulm |
| **Statistik und Biometrie** |
| **Dr. rer. nat. Johann M. Kraus**  Institut für Medizinische Systembiologie, Universität Ulm |

| **Protokollkomitee** |
| --- |
| **Prof. Dr. med. Thomas Seufferlein**  Klinik für Innere Medizin I, Universitätsklinikum Ulm |
| **Prof. Dr. rer. nat. Hans A. Kestler**  Institut für Medizinische Systembiologie, Universität Ulm |
| **Dr. med. Angelika Kestler**  Klinik für Innere Medizin I, Universitätsklinikum Ulm |
| **M.sc. Silke D. Kühlwein**  Institut für Medizinische Systembiologie, Universität Ulm |
| **Dr. rer. nat. Julian D. Schwab**  Institut für Medizinische Systembiologie, Universität Ulm |
| **M.sc. Robin Szekely**  Institut für Medizinische Systembiologie, Universität Ulm |
| **M.sc. Patrick Thiam**  Institut für Medizinische Systembiologie, Universität Ul |
| **Dr. rer. nat. Rolf Hühne**  Institut für Medizinische Systembiologie, Universität Ulm |
| **Dr. hum. biol. Axel Fürstberger**  Institut für Medizinische Systembiologie, Universität Ulm |
| **Franz Jobst**  Stabsstellen, Universitätsklinikum Ulm |

## 1.2 Studien Synopse

| **Studientitel** | NEMO – eine App für das Nebenwirkungs-Management in der Onkologie |
| --- | --- |
| **Kurzbezeichnung** | NEMO |
| **Studienkollektiv** | Patienten ab dem 18. Lebensjahr, die mit einer infusionalen Kombinationschemotherapie von mindestens zwei Chemotherapiesubstanzen (ohne Berücksichtigung von zusätzlicher zielgerichteter Therapie) behandelt werden. |
| **Phase I** | Machbarkeitsstudie/ Akzeptanzstudie der App |
| **Primäre Zielgröße** | Akzeptanz der Patienten eines neuen Kommunikationsmittels |
| **Phase II** | Verbesserung des Nebenwirkungsmanagements durch die App |
| **Primäre Zielgröße** | Reduktion der Nebenwirkungen um mindestens 1 Grad nach CTCAE zum Studienstart |
| **Sekundäre Zielgrößen** | - Beibehaltung der Chemotherapie-Dosen durch besseres Nebenwirkungsmanagement - Verbesserung der Lebensqualität durch besseres Nebenwirkungsmanagement |
| **Einschlusskriterien** | - Infusionale Kombinationschemotherapie mit mindestens zwei Chemotherapiesubstanzen mit / ohne zielgerichtete Therapien - Alter $\geq$ 18 Jahre - Schriftliches Einverständnis zur Teilnahme an der Studie |
| **Ausschlusskriterien** | - Schwere neurologische oder psychiatrische Störungen die eine Einwilligungsfähigkeit beeinträchtigen - Kein Einverständnis für die Registrierung, Datenübernahme und Handhabung der personenbezogenen Daten |
| **Studiendesign** | - Erste Phase Machbarkeitsstudie - Zweite Phase der Studie zur Erfassung von Nebenwirkungen und Lebensqualität bei Patienten, die eine infusionale Chemotherapie mit mindestens zwei Chemotherapiesubstanzen erhalten. |
| **Datenschutz** | Klinische Daten werden ausschließlich in pseudonymisierter Form verarbeitet. Für klinische Daten wird eine Patienten-ID als Identifizierungsnummer vergeben. Identifizierende Daten der Patienten werden vom klinischen Studienpartner (Klinik Innere Medizin I) verwaltet.  Probanden werden über die Verarbeitung ihrer personenbezogenen Daten und ihre Rechte aufgeklärt und willigen ausdrücklich in Teilnahme und Verarbeitung ein. |
| **Zentren** | Universitätsklinikum Ulm |

# 2. Ausgangssituation und Studienrationale

## 2.1 Hintergrund und Rationale

Bedingt durch die jeweiligen Tumortherapieprotokolle, aber auch auf Grund zeitlich begrenzter Ressourcen sehen Ärzte ihre ambulant behandelten Patienten nur in bestimmten zeitlichen Abständen. Patienten haben dann oft wichtige Informationen über aufgetretene Nebenwirkungen, Unverträglichkeiten, als auch über das allgemeine Befinden vergessen oder erachten diese Informationen als nicht relevant.

Insbesondere medikamentöse Kombinationstherapien in der Onkologie beinhalten häufig Wirkstoffe, bei denen Nebenwirkungen wie Übelkeit, eine Schleimhautentzündung (Mukositis), Durchfall (Diarrhö) oder eine Polyneuropathie auftreten können. Die Akzeptanz der Tumortherapie wird entscheidend durch das Auftreten, sowie der Dauer der Nebenwirkungen bestimmt. Besteht eine gute Verträglichkeit der Therapie durch die Reduktion von Nebenwirkungen, kommt es zu einer Verbesserung der Lebensqualität. Dies ist ein wichtiges Ziel einer Tumortherapie, in der auch psychoonkologische und soziale Bedürfnisse der Patienten mitberücksichtigt werden.

Für das standardisierte Protokollieren möglicher relevanter Nebenwirkungen und weiterer therapieassoziierten Informationen wurde eine App entwickelt, die es dem Patienten erleichtern soll, diese Nebenwirkungen festzuhalten.

In einer ersten Phase soll im Rahmen der vorliegenden Studie die Akzeptanz und Anwendbarkeit der App getestet werden. Sollte diese erste Phase mit positiven Ergebnissen abschließen, ist in einer zweiten Phase eine Studie geplant, die einen Effekt der Verwendung der App auf das Management von Nebenwirkungen und deren supportive Therapie, sowie das Beibehalten der ursprünglichen Chemotherapiedosis untersuchen soll.

# 3. Studiendesign

Im Rahmen dieser Studie sollen Patienten ab 18 Jahren, die eine Kombinationschemotherapie zur Behandlung einer Tumorerkrankung erhalten, eingeschlossen werden. Die Studiendauer ist auf 6 Monate angelegt.

Die diagnostischen Maßnahmen, wie auch die indizierten Untersuchungen werden dokumentiert und fließen in die Auswertung mit ein. Es wird weder in den Ablauf der onkologischen Behandlung, noch in anstehende Therapieentscheidungen eingegriffen. Die erhobenen Daten stehen lediglich dem behandelnden Arzt zur Verfügung und es ist ihm freigestellt, ob er sie in anstehende Therapieentscheidungen mit einbezieht.

## 3.1 Phase 1

### 3.1.1 Primärer Endpunkt

Die vorliegende Studie ist in zwei aufeinander folgende Phasen unterteilt. Der primäre Endpunkt der ersten Phase ist es zu untersuchen, ob neue Kommunikationsmittel, hier die Anwendung einer Smartphone-App zur Dokumentation von Therapienebenwirkungen und dem Befinden, Akzeptanz bei der angestrebten Patientengruppe findet. Als Grundlage dient ein Fragebogen zum allgemeinen Umgang mit Smartphones und zur Evaluierung der Anwendung. Die Ergebnisse dieser Auswertung ermöglichen es uns die Kriterien für die Rekrutierung von Patienten in Phase II unserer Studie (wie z.B. Alter) möglicherweise anpassen zu können.

## 3.2 Phase 2

### 3.2.1 Primärer Endpunkt

Sollte die erste Phase der Studie mit einem positiven Ergebnis enden, d.h. die Patienten nehmen die Smartphone-App an und kommen mit ihr zurecht, folgt eine zweite Phase der Studie. In dieser zweiten Phase ist der primäre Endpunkt zu untersuchen, ob durch Verwendung der App eine Reduktion der Nebenwirkungen um mindestens 1 Grad nach CTCAE im Vergleich zum Studienstart erreicht werden kann. Hierfür wird der tägliche Fragebogen zu den möglichen aufgetretenen Nebenwirkungen und dem Allgemeinbefinden herangezogen, der mit Hilfe der Smartphone-App selbständig von den Patienten beantwortet wird.

### 3.2.2 Sekundärer Endpunkt

In der zweiten Phase der Studie soll auf Grundlage des EORTC-QLQ-C30 Fragebogens^1^ untersucht werden, ob durch die Verwendung der App und ein verbessertes Nebenwirkungsmanagement auch die Lebensqualität der Patienten verbessert werden kann. Der EORTC-QLQ-C30 wird anhand des validierten EORTC-Handbuchs ausgewertet^2^.

Mittels des elektronischen Prüfbogens (eCRF = electronic Case Report Form) wird auch die verwendete Chemotherapie-Dosis im Verlauf der Studie dokumentiert. Auf Grundlage dieser Daten ist ein weiteres sekundäres Ziel der zweiten Phase zu untersuchen, ob es möglich ist die Chemotherapiedosen durch ein besseres Nebenwirkungsmanagement beizubehalten. Von einem positiven Effekt ist auszugehen, wenn die initial verabreichten Chemotherapie-Dosen über einen längeren Zeitraum bei Patienten, die die App verwenden, verabreicht werden können, im Vergleich zu einer Kontrollgruppe, die die App nicht verwendet.

### 3.2.1 Fragestellungen

- Erfassung von epidemiologischen, patientenbezogenen Basisdaten für die Auswertung der Machbarkeits- und Anwenderstudie zu neuen Kommunikationsmitteln im Therapiemanagement
- Erfassung von auftretenden Nebenwirkungen
- Korrelation von Eigen-/ Fremdwahrnehmung bezüglich dem Auftreten von Nebenwirkungen
- Korrelation zwischen dem Schweregrad von Nebenwirkungen und der Verwendung der App
- Korrelation Veränderung der Chemotherapie-Dosen und Verwendung der App
- Korrelation Lebensqualität und Nebenwirkungen

## 3.3 Detailliertes Design

### 3.3.1 Flow Chart

|  | Screening-Visite | Studieneinschluss/ Baseline-Visite | Erste Visite^1^ | Zweite Visite^1^ | Abschlussvisite^1^ |
| --- | --- | --- | --- | --- | --- |
| Prüfung Ein-/ Ausschlusskriterien | X |  |  |  |  |
| Patienteninformation | X |  |  |  |  |
| Einverständniserklärung | X |  |  |  |  |
| Festlegung des pseudonymisierten Patienten-ID |  | X |  |  |  |
| Fragebogen zur Handhabung der App |  |  |  |  | X |
| Fragebogen Lebensqualität |  | X |  |  | X |
| Fragebogen zu aufgetretenen Nebenwirkungen |  | X | X | X | X |
| eCRF |  | X | X | X | X |

^1^Visiten finden jeweils zusammen mit den alle 2 Monate stattfindenden CT- Staging Untersuchungen über einen Zeitraum von insgesamt 6 Monaten statt.

### 3.3.2 Dokumentation von Ereignissen während der Studie

Ereignisse, die eine Änderung der Therapie bedürfen, wie Deeskalation einer initial mindestens Zweifachchemotherapie auf eine Monotherapie aufgrund einer aufgetretenen Toxizität, progredienten Tumorerkrankung, einer Deeskalation auf eine Maintenance-Therapie oder Therapiepause bei stabiler Tumorerkrankung werden in der App dokumentiert. Bei Therapieende der medikamentösen Therapie vor Ablauf der Studie wird diese ebenfalls dokumentiert und die Patienten erhalten die Fragebögen der Abschlussvisite.

### 3.3.3 Machbarkeit/ Akzeptanz der App

Ein primärer Endpunkt der Beobachtungsstudie befasst sich mit der Anwendbarkeit der App und der Akzeptanz bei den Patienten. Hierfür wurde ein Fragebogen erstellt, der die bisherigen Smartphone-Kenntnisse der Patienten, sowie die Handhabung und Funktionalität der Anwendung der App erfragt.

### 3.3.4 Lebensqualität

Ein Bogen zur Lebensqualität der Patienten wird begleitend zu Studienbeginn und nach 6 Monaten erfasst. Dabei handelt es sich um den validierten Fragebogen EORTC QLQ-C30^1^.

#### 3.3.4.1 Definierte Fragestellung zur Lebensqualität

- Generelle Erfassung von Veränderungen (Verschlechterung / Verbesserung) der Lebensqualität
- Ausschluss von psychischen Faktoren auf das Auftreten von Nebenwirkungen

### 3.3.5 Epidemiologische Datenerfassung

Der Gebrauch von Smartphones ist in der heutigen Gesellschaft stark angestiegen^3^. Allerdings sind Unterschiede zwischen den Altersgruppen zu erkennen. Während Altersgruppen, die mit dieser Entwicklung aufgewachsen sind verstärkt auf digitale Unterstützer setzen, ist dieser Trend bei einer Altersgruppe über 60 Jahre weniger stark ausgeprägt^4^. Um den Einfluss des digitalen Lebensstils auf die Akzeptanz der Anwendung abschätzen zu können, wurden in den Fragebogen zur NEMO-Smartphone-App zusätzliche Fragen integriert, die auf die allgemeine Verwendung von Smartphones abzielen.

Die Erfassung der epidemiologischen Daten erfolgt zum Zeitpunkt des Studienabschlusses anhand des Fragebogens zur NEMO-Smartphone-App, der von den Patienten selbständig ausgefüllt wird. Die Verarbeitung und Speicherung der Daten erfolgt in pseudonymisierter Form unter Verwendung des NEMO-Identifiers.

### 3.3.6 Klinische Datenerfassung

Die Erfassung klinischer Daten erfolgt zum Studieneinschluss und während der Studienvisiten (siehe 3.3.1). Die erhobenen Daten sollen in den vorgesehenen Datenblättern des elektronischen Case Report Form (eCRF) übertragen werden. Die Verarbeitung und Speicherung der Daten erfolgt in pseudonymisierter Form unter Verwendung des NEMO-Identifiers.

### 3.3.7 Zeitpunkte zur Erfassung von Fragebögen

Die Erhebung des Fragebogens zur Smartphone-App erfolgt in der ersten Phase der Studie nach Studienabschluss anhand eines Fragebogens in Papierform, der von den Patienten selbständig ausgefüllt wird.

Die Erhebung des Fragebogens zur Lebensqualität erfolgt bei der Baseline Visite und nach Abschluss der Studie. Es wird ein Fragebogen in Papierform ausgehändigt, der von den Patienten selbständig ausgefüllt wird.

Die Verarbeitung und Speicherung der Daten erfolgt in pseudonymisierter Form unter Verwendung des NEMO-Identifiers.

### 3.3.8 Statistik

#### 3.3.8.1 Biometrie

Im Rahmen der Pilotstudie soll eine Gesamtfallzahl von 30 Probanden aufgeteilt in drei Altersgruppen im Hinblick auf die Bedienbarkeit der App untersucht werden. Basierend auf diesen Erkenntnissen ist eine zweite Studie unter Berücksichtigung möglicher Stratifikationen bezüglich des Alters und der Technikaffinität der Nutzer geplant.

In dieser zweiten Phase soll untersucht werden, ob es zu einer Reduktion der Nebenwirkungen während der onkologischen Therapie kommt, wenn App-Nutzer mit einer Kontrollgruppe verglichen werden.

Zur Beantwortung dieser Fragestellung wurde eine Stichprobengröße mit der statistischen Software G*Power ermittelt. Hierbei wurde angenommen, dass sich eine Effektgröße von 1 aus einer Verbesserung der Nebenwirkungsgrade auf der Likert-Skala um 1 ergibt. Bei einem zweiseitigen Gruppenvergleich (Signifikanzniveau = 0,05) und einer Teststärke von 80% ergibt sich hieraus eine gesamte Stichprobengröße von n = 36 (Abb. 1) aufgeteilt in zwei Gruppen à 18 Probanden.

Abbildung 1: Fallzahlabschätzung basierend auf G*Power

#### 3.3.8.2 Statistische Auswertung

Um die Validität bekannter Gruppen zu beurteilen sowie Unterschiede im Schweregrad auftretender Nebenwirkungen zwischen NEMO-Benutzern und Patienten mit Standard-Berichterstattung bewerten zu können, soll ein Wilcoxon-Mann-Whitney-Test angewendet werden.

Die Bewertung mittels NEMO dokumentierten Nebenwirkungen soll mit Hilfe einer Gesamtpunktzahl erfolgen. Hierzu wird die Likert-Skala herangezogen, über die jede aufgetretene Nebenwirkung bewertet wird. Die Gesamtbewertung ergibt sich aus einer zusammengefassten Punktzahl einer gewichteten Summe der Fragebogenelemente mit der maximalen Anzahl an zu beantwortenden Elementen. Zusätzlich soll die Spearman Korrelation angewendet werden, um die konvergente Validität zwischen jeder Frage des NEMO-Fragebogens und der Lebensqualität zu bewerten.

Ob die Anwendung von NEMO einen Einfluss auf das Nebenwirkungsmanagement hat, soll mittels des Jonckheere-Terpstra Tests^5^ analysiert werden. Hierfür werden die Änderungen des Fragenbewertungen zwischen den Therapiesitzungen verglichen.

# 4. Patienten

Die Beantwortung der im Rahmen der NEMO Studie gestellten Fragebögen kann nur bei entsprechender Auswahl und Einschluss der Patienten erfolgen. Entsprechend sind unten angegebene Kriterien zu erfüllen. Die Wahrung der Ein- und Ausschlusskriterien obliegt dem lokalen Prüfarzt. Bei Unklarheiten sollte Kontakt mit dem Studienkoordinator aufgenommen werden.

## 4.1 Einschlusskriterien

- Infusionale Chemotherapie mit mindestens zwei Chemotherapiesubstanzen mit / ohne zielgerichtete Therapien
- Alter $\geq$ 18 Jahre
- Schriftliches Einverständnis zur Teilnahme an der Studie

## 4.2 Ausschlusskriterien

- Schwere neurologische oder psychiatrische Störungen, die eine Einwilligungsfähigkeit beeinträchtigen
- Kein Einverständnis für die Registrierung, Datenübernahme und Handhabung der personenbezogenen Daten

# 5. Dauer der Studie/ Studienabbruch

Die Datenerhebung im Rahmen der Studie erfolgt über 6 Monate. Ein Zurückziehen des Einverständnisses zur Studie ist durch den Patienten jederzeit ohne Angabe von Gründen möglich.

# 6. Datenmanagement

## 6.1 Datenschutz

Alle im Rahmen der Studie verarbeiteten personenbezogenen Daten unterliegen den geltenden datenschutzrechtlichen Bestimmungen. Personenbezogene Daten werden mit Ausnahme von Alter und Geschlecht weder vom Leiter der klinischen Studie noch von den beteiligten Prüfern weitergegeben. Die Weitergabe von klinischen Daten und die Speicherung zur Auswertung erfolgt nur in Verbindung mit einem eindeutigen Pseudonym (NEMO-Identifier). Nach Abschluss der Studie werden die Studienunterlagen gemäß den Bestimmungen des Datenschutzes archiviert. Personen, denen ein Zugriff auf die Daten ermöglicht wird, unterliegen der Schweigepflicht. Eine unbefugte Weitergabe der Daten ist ihnen untersagt.

Alle Daten werden in Form von elektronischen CRFs erfasst und in eine Datenbank eingespeist. Das Datenmanagement wird vom Institut für Medizinische Systembiologie der Universität Ulm durchgeführt. Die Datenauswertung erfolgt ebenfalls im Institut für Medizinische Systembiologie der Universität Ulm.

Die Daten werden unter den Kriterien von Good Clinical Practice (GCP) bzw. Good Epidemiological Practice (GEP) verwendet.

Die klinischen Daten werden ausschließlich in pseudonymisierter Form verarbeitet. Die Stammdaten der Patienten werden vom klinischen Studienpartner (Klinik für Innere Medizin I, Universitätsklinikum Ulm) verwaltet.

## 6.2 Patienteninformation und Einwilligungserklärung

Jeder Patient ist vor Aufnahme in die NEMO Studie vom behandelnden Arzt über Ziel der Studie, Art und Umfang der Dokumentation aufzuklären. Ohne die schriftliche Einwilligung des Patienten für die Verarbeitung seiner Daten im Rahmen von NEMO darf er nicht in diese Studie eingeschlossen werden. Die Einwilligungserklärung muss in zweifacher Ausführung vollständig ausgefüllt und unterschrieben werden. Je ein Exemplar verbleibt beim Patienten und eines beim aufklärenden Arzt. Der Schutz der Daten des Patienten wird immer und unbedingt gewährleistet. Die im Rahmen von NEMO erhobenen Patientendaten werden in pseudonymisierter Form im eCRF dokumentiert, d.h. nur mit einer Kennziffer (NEMO-Identifier) und ohne Nennung des Namens, der Initialen, des Geburtsdatums oder der Anschrift des Patienten. Im Falle einer Veröffentlichung der Studienergebnisse dürfen die personenbezogenen Daten nur in anonymisierter Form verwendet werden. Einsicht in personenbezogene Daten dürfen nur autorisierte und zur Verschwiegenheit verpflichtete Beauftragte des Auftraggebers, sowie die zuständigen Überwachungsbehörden nehmen, soweit dies für die Überprüfung der ordnungsgemäßen Durchführung der Studie erforderlich ist.

Das für die Übertragung der Daten aus der Patientenakte zuständige medizinische Personal ist über seine datenschutzrechtliche Verantwortlichkeit aufzuklären.

## 6.3 Datenerfassung

Die Dokumentation erfolgt durch den behandelnden Arzt webbasiert in das eCRF, mit Ausnahme der als Papierversion vorliegenden Patientenfragebögen zur NEMO-Smartphone App und der Lebensqualität. Die validierte eCRF-Webapplikation befindet sich auf einem gesicherten und zugangsbeschränkten Server des Instituts für Medizinische Systembiologie der Universität Ulm. Programmierte Validierungen überprüfen bei Eingabe die Einträge in die Webapplikation auf Plausibilität und Fehlerfreiheit und generieren automatisch entsprechende Rückmeldungen. Offene Rückfragen sind im Status aktuell ersichtlich und nachzubearbeiten. Offene Rückfragen werden regelmäßig im System verfolgt und dem Zentrum kommuniziert. Die Bearbeitung von Rückfragen soll über das eCRF-Modul durch das Zentrum online erfolgen.

Die Patientenfragebögen zur Smartphone-App und der Lebensqualität sollen handschriftlich vom Patienten mit einem Kugelschreiber ausgefüllt und anschließend elektronisch in eine Datenbank eingelesen werden.

## 6.4 NEMO Registrierung

Alle Patienten, die an der Studie teilnehmen, erhalten obligat einen NEMO-Identifier, der als Pseudonym dient. Diese Pseudonymisierung dient der Nachvollziehbarkeit und Vergleichbarkeit der erhobenen Daten unter Wahrung des Datenschutzes.

### 6.4.1 Beschreibung des Pseudonymisierungsprozess

Die zur App gehörende Desktop Anwendung des Arztes liefert das Pseudonym NEMO-Identifier für den teilnehmenden Probanden. Dabei handelt es sich um ein isoliertes System, das unabhängig von der Smartphone-App ist.

Für die Pseudonymisierung und das Identitätsmanagment der Patientendaten wird gPAS® (generic Pseudonym Administration Service)^6^ verwendet. Hierbei handelt es sich um einen Open-Source Pseudonymisierungsdienst, der im Rahmen des DFG-geförderten Projekts „MOSAIC“ entwickelt wurde und bereits in einigen Projekten im Einsatz ist. Das Domänenkonzept, sowie die freie Definition von Alphabeten erlaubt unterschiedliche Pseudonyme je Datenquelle, Anwendungskontext oder Standort zu generieren. Zusätzlich kann bestimmt werden, wie Fehlertolerant das generierte Pseudonym sein soll^6,7^.

# 7. Qualitätssicherung

## 7.1 Standardisierung, Validierung und Monitoring

Das Monitoring wird durch den Leiter des Klinischen Krebsregisters übernommen. Es werden u.a. die folgenden Eckdaten überprüft: Demographische Daten, Ein- und Ausschlusskriterien, Einwilligungserklärung, Abbruch der Studie. Da das Studienzentrum in der Durchführung von klinischen Studien sehr erfahren ist, werden für diese Studie keine eigenen Prüfarztschulungen durchgeführt, sondern das Studienzentrum trägt für die Schulung der eigenen Mitarbeiter Sorge. Folgender Qualitätsindikator wird für den Studienablauf festgelegt: Einhaltung der Auswahlkriterien. Der behandelnde Arzt erklärt per Vertragsunterschrift sein Einverständnis, alle Daten dem zuständigen Monitor im Falle einer Überprüfung zugänglich zu machen. Das Vorliegen einer von dem Patienten unterzeichneten Einverständniserklärung ist Voraussetzung für die Übermittlung pseudonymisierter Daten und die Teilnahme an der Erhebung der Patientendaten. Diese muss durch den aufklärenden Arzt nach Überprüfung der Ein- und Ausschlusskriterien garantiert werden. Bei Unsicherheiten bezüglich der Ein- und Ausschlusskriterien im speziellen Fall soll der zuständige Studienkoordinator kontaktiert werden.

# 8. Gesetzliche und administrative Regelungen

In der Studie werden die Empfehlung zu Good Clinical Practice (s. ICH-GCP: International Conference on Harmonisation – Good Clinical Practice), gültig seit dem 17.01.1997, berücksichtigt. Ebenso gelten die Empfehlungen zu [Good *Epidemiological* Practice](https://www.google.com/search?client=safari&rls=en&q=Good+Epidemiological+Practice&spell=1&sa=X&ved=0ahUKEwi_jK3W-ZDkAhUOUlAKHVFYBloQkeECCCwoAA) (https://www.dgepi.de/assets/Leitlinien-und-Empfehlungen/Recommendations-for-good-Epidemiologic-Practice.pdf).

## 8.1 Ethikkommission

Vor Beginn der Studie wird die für den ärztlichen Leiter von NEMO zuständige, nach Landesrecht gebildete Ethikkommission beratend hinzugezogen. Ein Studieneinschluss ist erst nach Erhalt eines positiven Ethikvotums für die Studie möglich. Dem Patienten wird eine schriftliche Patienteninformation zu Datenschutz und Einsichtnahme in seine Patientenakte sowie eine Einwilligungserklärung zur Unterschrift vorgelegt. Die schriftliche Einwilligung ist Voraussetzung für den Einschluss des Patienten in diese Studie.

## 8.2 Aufklärung und Einwilligung der Probanden

Vor Aufnahme in die Studie wird jeder Proband vom behandelnden Arzt über Wesen, Ziele, erwartete Vorteile und mögliche Risiken der Studie informiert. Jeder Proband muss, nach ausreichender Bedenkzeit, seine schriftliche Einwilligung zur Teilnahme an der Studie erklären. Dem Probanden wird dabei ausreichend Zeit und Gelegenheit gegeben, um vor der Einleitung der Studienmaßnahmen über seine Teilnahme zu entscheiden und offene Fragen zu klären. Die Einwilligungserklärung wird von dem Probanden und von dem behandelnden Arzt unterzeichnet. Ist der Proband nicht in der Lage, eigenhändig zu unterschreiben, muss ein Zeuge die erfolgte mündliche Aufklärung durch Unterschrift bestätigen.

Ein Muster der Probandeninformation und Einwilligungserklärung sind als Anhang beigefügt.

Probandeninformation und Einwilligungserklärung liegen in zweifacher Ausfertigung vor. Ein Exemplar verbleibt beim Prüfer, das andere ist dem Probanden auszuhändigen.

## 8.3 Finanzierung

Eine Finanzierung von NEMO erfolgt aus Mitteln des Zentrums für Innovative Versorgung (ZIV) im Rahmen der Digitalisierungsoffensive des Landes Baden-Württemberg.

## 8.4 Verwendung, Speicherung und Weitergabe von Daten

Die Probanden werden darüber informiert, dass ihre krankheitsbezogenen Daten in pseudonymisierter Form gespeichert und für wissenschaftliche Auswertungen verwendet werden. Die Probanden haben das Recht, über die gespeicherten Daten informiert zu werden. Für die Probanden ist es ersichtlich, wer mit welchem Fokus Einsicht in seine Daten erhält. Der Auftraggeber ist für die Archivierung der Studienunterlagen über einen Zeitraum von mindestens 10 Jahren verantwortlich. Gemäß der MBOÄ (Musterberufsordnung für Ärzte) ist der teilnehmende Arzt verpflichtet, die Identifizierungsliste und die Dokumentation der im Rahmen der nicht-interventionellen Studie durchgeführten Daten mindestens 10 Jahre lang zu archivieren.

## 8.5 Publikation

Nach Auswertung der Daten ist eine zeitnahe und zügige Publikation der Ergebnisse vorgesehen. Es wird eine Publikation zur Beantwortung der Hauptfrage geben (unabhängig davon, wie die Ergebnisse ausfallen). Als Autoren sind beteiligt: Das Studienkomitee sowie die Hauptprüfer des Studienzentrums, die durch Einschluss von mehr als 10% der Gesamtzahl der Studienteilnehmer an der Rekrutierung maßgeblich mitgewirkt haben. Weitere Autoren werden nach ihrem wissenschaftlichen Input zur Auswertung bzw. der Manuskripterstellung aufgenommen. Neben der Hauptfragestellung sind weitere Publikationen vorgesehen, darunter die Publikation der explorativen Auswertung zur Reduktion von Nebenwirkungsgraden der zweiten Phase dieser Studie. Die Publikationen werden in Absprache mit der Studienleitung erstellt. Patientendaten werden im Rahmen der Publikation anonymisiert dargestellt. Kongressbeiträge können im Rahmen von Zwischenauswertungen/Endauswertungen in Absprache mit der Studienleitung erfolgen.

**Literaturverzeichnis**

1. Aaronson, N. K. *et al.* The European Organization for Research and Treatment of Cancer QLQ-C30: A Quality-of-Life Instrument for Use in International Clinical Trials in Oncology. *JNCI: Journal of the National Cancer Institute* **85**, 365–376 (1993).

2. Fayers, P. *et al.* *The EORTC QLQ-C30 Scoring Manual (3rd Edition)*. (European Organisation for Research and Treatment of Cancer, 2001).

3. Joe, J. & Demiris, G. Older adults and mobile phones for health: A review. *Journal of Biomedical Informatics* **46**, 947–954 (2013).

4. Pöpsel, F. Zukunft ist heute. *#smart gesund* (2019).

5. Jonckheere, A. R. A Distribution-Free k-Sample Test Against Ordered Alternatives. *Biometrika* **41**, 133–145 (1954).

6. Geidel, L., Bahls, T. & Hoffmann, W. A generic pseudonymization tool as a module of Central Data Management for medical research data (Ein generisches Pseudonymisierungswerkzeug als Modul des Zentralen Datenmanagements medizinischer Forschungsdaten). in 245–246 (2013).

7. Insitut für Community Medicien, Abt. VC, Universitätsmedizin Greifswald. *Pseudonymverwaltung mit gPAS*. (2013).
