# Supplementary material for: Digitalization of adverse event management in oncology to improve treatment outcome—A prospective study protocol
Source: PLoS One. 2021 Jun 4;16(6):e0252493. doi: 10.1371/journal.pone.0252493 (PMC8177479; doi:10.1371/journal.pone.0252493)
Supplement: S1 Table — The grades refer to the severity of the occurred adverse effect. The CTCAE scoring includes grade 1 to 5. We modify this scoring including grade 0 (= no side effect) and stopped our scoring at grade 3. This procedure is based on the fact that our app is designed for outpatients, thus excluding life-threatening consequences (grade 4) or death (grade 5). The meaning of each grading is in accordance with (CTCAE v5.0 Clean, Tracked, and Mapping Document) and was translated into German. (PDF) [file pone.0252493.s004.pdf]

S 1 Table. Smartphone questionnaire for chemotherapies.

	Smartphone questionnaire	Grade 0	Grad 1	Grad 2	Grad 3
General	How are you?	very good	good	moderate	poor
	Did you excercise today?	yes, more than 30 minutes	no, mostly no exercise		
	Was today something special?	Please comment			
	Did you require the demand medication?	no	yes		
CTCAE	How often did you go to the bathroom with diarrhea today?	not at all	1-3 times	4-6 times	more than 6 times
	How many times did you vomit today?	not at all	1-2 times	3-5 times	more than 5 times
	Do you have any pain?	no	few	moderate	severe
	Do you have pain when you eat?	no pain	flush, but no pain	moderate pain, normal eating	severe pain, limited eating
	How did you eat today?	normal, with appetite	without appetite, but normal	less than normal	very little to nothing
	Do you have sensibility disorders?	no	yes, sporadic	yes, mild and persistent	yes, severe and persistent
	Are you excessively tired?	no	yes, but resting helps	yes, and it is not getting better by resting	yes, and I require permanent help
	What is your weight? (Used to calculate weight loss)	<5% weight loss	5-10% weight loss	10-20% weight loss	>20% weight loss

Remark: The grades refer to the severity of the occurred adverse effect. The CTCAE scoring includes grade 1 to 5. We modify this scoring including grade 0 (= no side effect) and stopped our scoring at grade 3. This procedure is based on the fact that our app is designed for outpatients, thus excluding life-threatening consequences (grade 4) or death (grade 5). The meaning of each grading is in accordance with (CTCAE v5.0 Clean, Tracked, and Mapping Document) and was translated into German.
