# Supplementary material for: Digitalization of adverse event management in oncology to improve treatment outcome—A prospective study protocol
Source: PLoS One. 2021 Jun 4;16(6):e0252493. doi: 10.1371/journal.pone.0252493 (PMC8177479; doi:10.1371/journal.pone.0252493)
Supplement: S3 Table — (PDF) [file pone.0252493.s006.pdf]

S 3 Table. Questions for smartphone feasibility.

<table>
<tr><th></th><th>Question</th><th colspan="4">Response options</th></tr>
<tr><td rowspan="6">General</td><td>Age</td><td><55 years</td><td colspan="2">55 - 77 years</td><td>>77 years</td></tr>
<tr><td>Sex</td><td colspan="2">male</td><td colspan="2">female</td></tr>
<tr><td>Handedness</td><td colspan="2">right-handed</td><td colspan="2">left-handed</td></tr>
<tr><td>How often do you use smartphones?</td><td>daily</td><td colspan="2">several times a week</td><td>rarely</td></tr>
<tr><td>How do you judge your smartphone handling?</td><td>very good</td><td>good</td><td>medium</td><td>minor</td></tr>
<tr><td>Which operating system do you use?</td><td>Android</td><td>iOS</td><td colspan="2">other</td></tr>
<tr><td rowspan="10">Smartphone app</td><td>Is the app easy to read?</td><td>very good</td><td>good</td><td>medium</td><td>poorly</td></tr>
<tr><td>Are the questions of the app comprehensibly?</td><td>very good</td><td>good</td><td>medium</td><td>poorly</td></tr>
<tr><td>Is the app clearly laid out?</td><td>very good</td><td>good</td><td>medium</td><td>poorly</td></tr>
<tr><td>Is the app easy to use?</td><td>very good</td><td>good</td><td>medium</td><td>poorly</td></tr>
<tr><td>Is the time to answer the questions of the app reasonable?</td><td>very good</td><td>good</td><td>medium</td><td>poorly</td></tr>
<tr><td>Was the app a support for the communication with your physician?</td><td colspan="2">yes</td><td colspan="2">no</td></tr>
<tr><td>Would you recommend the app to others?</td><td colspan="2">yes</td><td colspan="2">no</td></tr>
<tr><td>Was there a problem in the functionality of the app?</td><td colspan="4">Please enter a text</td></tr>
<tr><td>Could the app display your complaints?</td><td colspan="4">Please enter a text</td></tr>
<tr><td>Any further comments? Would you like to tell us something about your experience with the app or make suggestions for improvement?</td><td colspan="4">Please enter a text</td></tr>
</table>
