# Supplementary material for: Digitalization of adverse event management in oncology to improve treatment outcome—A prospective study protocol
Source: PLoS One. 2021 Jun 4;16(6):e0252493. doi: 10.1371/journal.pone.0252493 (PMC8177479; doi:10.1371/journal.pone.0252493)
Supplement: Attachment — Submitted filename: ResponseToReviewers.pdf [file pone.0252493.s008.pdf]

Response to Editors and Reviewers

We thank the reviewers and the academic editor for their valuable comments. We believe that this further increased the quality of the manuscript. Please find below our point to point responses.

Editors / Journal Office:

1. Please ensure that your manuscript meets PLOS ONE's style requirements, including those for file naming. The PLOS ONE style templates can be found at

https://journals.plos.org/plosone/s/file?id=wjVg/PLOSOne_formatting_sample_main_body.pdf and https://journals.plos.org/plosone/s/file?id=ba62/PLOSOne_formatting_sample_title_authors_affiliations.pdf

Answer 1: Yes we adhere to these guidelines.

2. Thank you for stating in your ethics statement on your online submission form

"Ethics committee Ulm University, Ulm, Germany; Proposal no. 406/19: "This concludes the evaluation by the research ethics committee of the University of Ulm with a positive opinion. Consent was obtained in written form." For studies reporting research involving human participants, PLOS ONE requires authors to confirm that this specific study was reviewed and approved by an institutional review board (ethics committee) before the study began. Please clarify whether this study protocol was specifically approved by your ethics committee.

Answer 2: Yes, this study protocol was specifically approved by the ethics committee. The study has not yet begun.

3. The text of the manuscript states that the protocol for phase II was approved by your ethics committee. Please clarify if the protocol for phase I was also approved by the committee.

Answer 3: Yes, this study, comprised of two parts, was approved by the ethics committee as stated before. We rephrased that in the manuscript, so not to confuse it with phase I and II trails, which this study is not.

4. Please provide additional details regarding participant consent. In the ethics statement in the Methods and online submission information, please ensure that you have specified whether consent was informed.

Answer 4: Yes, we do.

5. To meet our data availability standards, please provide a link for the smartphone/desktop application described in this study, or provide detailed instructions as to how another researcher could access it.

Answer 5:
Nemo smartphone app: https://tinyurl.com/nemoappios and https://tinyurl.com/nemoappandroid
Desktop-App: https://github.com/sysbio-bioinf/NEMODesktop

6.Please amend either the abstract on the online submission form (via Edit Submission) or the abstract in the manuscript so that they are identical.

Answer 6: We changed this accordingly.

Reviewer 1:

The authors present the protocol of a study obtaining Patient-Reported-Outcomes (PRO) and especially CTCAE criteria. The manuscript is thouroughly written (please note that I am not a native speaker myself). The different sections of the manuscript do not leave much room for improvement. The proposed study is feasible, methods are adeqante and most important the research question and the study is meaningful and will provide patient relevant data.
I only have a few comments.

Comment 1:
I checked for accuracy conconrinng to the equator-network guidelines. Here, the title should be in line with the recommendations. I would suggest adding a short sentence as second part ouf the titel as "A protocol for a non-randomized prospective feasibility study.

Response 1:
Thank you for raising this issue. According to your suggestion, we added a second part to the initial title..

Comment 2:
In the discussion and part of the introduction the authors are not precide what really is their underlying aim. For me, it is specifically in screening and early detection of CTCAE criteria. This can be stressed as one of the unique selling points of this study. You may refer as an example to another feasibility study (1) to underline that other studies have merely looked at PROs (a very vast range), but your unique selling point is, again, the adherance to CTCAE criteria.
1 Benze G et al. PROutine: a feasibility study assessing surveillance of electronic patient reported outcomes and adherence via smartphone app in advanced cancer. Ann Palliat Med. 2019 Apr;8(2):104-111.)

Response 2:
Thank you for your comment. Based on your comment, we now emphasise this already in the abstract.
Here, the first part (feasibility study) is intended to identify barriers in the use of the new designed application. The obtained results will enable us to possibly amend the criteria for recruiting patients (e.g. regarding age) to assess if the use of the smartphone application for adverse event tracking improves patients' safety and outcomes.
Besides, we agree that the study of Benze et al. is an interesting report of the feasibility of smartphone based patient reported outcomes which we now include in our citations.

Comment. 3:
Please note, that I have not checked manuscript- and references according to the journal style, but leave this to the editorial office.

Response 3:
Please see our response 1 to Journal Office above.

Reviewer 2:

Important note: This review pertains only to 'statistical aspects' of the study and so 'clinical aspects' [like medical importance, relevance of the study, 'clinical significance and implication(s)' of the whole study, etc.] are to be evaluated [should be assessed] separately/independently. Further please note that any 'statistical review' is generally done under the assumption that (such) study specific methodological [as well as execution] issues are perfectly taken care of by the investigator(s). This review is not an exception to that and so does not cover clinical aspects {however, seldom comments are made only if those issues are intimately / scientifically related & intermingle with 'statistical aspects' of the study}. Agreed that 'statistical methods' are used as just tools here, however, they are vital part of methodology [and so should be given due importance].

COMMENTS: I highly appreciate the study [particularly the objective [Early detection and treatment of adverse events in oncological treatment to improve patients' safety and outcomes]. However, I have few small {really very 'minor'} queries. Those are given below. Hope, you will find them worth considering.

Comment 1:

1. Although your 'Article Type: Registered Report Protocol' it is not indicated anywhere that it is a 'Protocol'. Preferably the word 'Protocol' should appear in title itself. Mention at least in 'abstract' is expected.

Response 1:

Thank you for pointing this out. Now the second part of the title highlights that this manuscript is a prospective study protocol. Besides, we clarify this issue within the abstract. Therefore, we reduced the description of the smartphone-application itself and focus now on the prospective clinical trial.

Comment 2:

2. Since 'Phase I' is only to assess the feasibility of the application why patients divided into three age groups (<55years, 55-75years, >75years). Do you wish to assess the feasibility of the application for different age groups? You also say that 'equal-sized' groups. How was that insured? Use of 'quota' sampling may be mentioned.

Response 2:

The purpose of first phase is to assess the general feasibility of the smartphone application. Here, we evaluate if a certain age group does not cope with the application. If this would be the case, we would amend the second phase and add an age restriction to allow randomization of the arms ("standard-care" vs. "smartphone-users + standard care"). To clarify this procedure, we added as suggested by you that we applied quota sampling.

Comment 3:

3. According to 'abstract-methods' phase II, will compare the impact of smartphone reported adverse events regarding applied therapy doses and quality of life to those of patients receiving standard care. Here, 36 patients will be randomly assigned to each of the two groups. Which two groups is not described there? It is clear only from figure-2 that 'Patients in group 1: Smartphone questionnaire + standard care' and Patients in group 2 (control) : Standard care'.

Response 3:

Thank you for pointing this out. We changed the text accordingly.

Comment 4:

4. In figure-2 Y-axis heading is 'Total Sample Size' but generally sample size is given 'per arm'. I request you to kindly confirm {May please refer to line 124}. Figure-2 is 100% correct, but modified/ adjusted/derived.

Response 4:
We enlarged the figure legend of figure 2 focusing on the two arms both including 18 participants leading in total to a sample size of 36 participants.

Comment 5:
5. Supporting information files are not named as 'S1 Table. Smartphone questionnaire for chemotherapies', 'S2 Table. Smartphone questionnaire for immunotherapies' and 'S3 Table. Feasibility analysis' as indicated in manuscript. Therefore, 'S3 Table. Feasibility analysis' is not available [which is important for me].

Response 5:
Thank you for pointing this out. This happened by mistake. The tables of the Supplement are of course Table S1 to S3.

Comment 6:
6. Refer to section 'Biometry - sample size estimation' as well as to lines 148-49 which says regarding Wilcoxon-Mann-Whitney test [Wilcoxon-Mann-Whitney test will be used to assess known-groups validity, as well as 148 to compare differences between NEMO users and patients with standard reporting procedure concerning the severity of occurred adverse events] however, note that 'Wilcoxon's Rank Sum' test and 'Mann-Whitney U' test are separate tests [though equivalent]. There is no test by all three together {test called 'Wilcoxon-Mann-Whitney test'} in my knowledge. Kindly check. (scientist Wilcoxon had given one more test called 'Wilcoxon's Signed Ranked test' which is for paired samples)

Response 6:
We agree with the reviewer that the naming of this test was mixed throughout the manuscript. According to the usage in “Practical Nonnparametric Statistics” by Conover, we use Mann-Whitney test throughout. All misleading namings were changed accordingly.

Comment 7:
7. If feasibility study is already completed ['S3 Table. Feasibility analysis' indicates so] then why 'Phase I' [which you say is to assess the feasibility of the application] because anyway in this article few sections are only for phase II [though not specified so (which is not desirable, I guess) ex.: section 'Biometry - sample size estimation']. Inclusion of 'Phase I' may increase confusion.

Response 7:
The whole study has not yet begun. The wording here was misleading. We now clarify this in the text.

Comment 8:
8. Further comment on (more account but very briefly) on 'Jonckheere-Terpstra test' is required, I guess (line 157). Why do you need 'A distribution-free k-sample test against ordered alternatives'?

Response 8:
We thank the reviewer for raising this point. To clarify the use of the Jonkheere-Terpstra test we changed the description: The impact of the side effect management will be analyzed in the second phase by comparing changes in an item's scoring between the reevaluation steps during tumor therapy. For this analysis of responsiveness we plan to test for monotonically decreasing scores within the set of items. This comparison will be made using a Jonckheere-Terpstra test.

Reviewer 3:

This an interesting area of research.

Comment 1:

1. It is not clear from the title that this paper describes the design of a future trial using the new data collection app that the authors have designed. The title and abstract should be changed to make this more obvious.

Response 1:

Thank you for this remark. We adapted the title and hopefully sharpened the abstract in this direction.

Comment 2:

2. As noted above, the paper describes a future trial. However, it also describes a tool for collecting adverse events using a smartphone app. This app is novel but its development is not described in enough detail. How does AE collection using the app compare with standard AE data collection? Do patients and clinicians like the app? How exactly did they participate in the app development?

Response 2:

The app development was in close cooperation with clinicians as can be observed from the author affiliations. A. Kestler and T. Seufferlein are senior physicians actively treating tumor patients. Furthermore, TS is also president of the German Cancer Society. App development started with an idea from AK and discussions with HK. During app development different not initially envisioned components like flexibility regarding HIS systems to also ensure locally treating physicians an access to the data, patient empowerment by only transferring data via QR-codes, or adverse events for targeted therapies, etc. were implemented through intensive discussions of participating computer scientists and clinicians.

Comment 3:

3. The figures showing the app and the "clinician dashboard" are in German. Since most readers of the medical literature may not understand German, the authors need to consider an english language version of the figures.

Response 3:

Thank you for pointing this out. Based on your comment, we modified the figures accordingly.
