# Supplementary material for: Digitalization of adverse event management in oncology to improve treatment outcome—A prospective study protocol
Source: PLoS One. 2021 Jun 4;16(6):e0252493. doi: 10.1371/journal.pone.0252493 (PMC8177479; doi:10.1371/journal.pone.0252493)
Supplement: Attachment — Submitted filename: renamed_400fd.docx [file pone.0252493.s007.docx]

**COMMENTS – Manuscript PONE-D-20-29066**

Title: “Digitalization of adverse event management in oncology to improve treatment outcome”

**Important note: This review pertains only to ‘statistical aspects’ of the study and so** **‘****clinical aspects’ [like medical importance,** **relevance of the study,** **‘clinical significance and** **implication(s)’ of the whole study, etc.] are to be evaluated [should be assessed] separately/independently. Further please note that any ‘statistical review’ is generally done under the assumption that (such) study specific methodological [as well as execution] issues are perfectly taken care of by the investigator(s). This review is not an exception to that and so does not cover clinical aspects {however, seldom comments are made only if those issues are intimately / scientifically related & intermingle with ‘statistical aspects’ of the study}. Agreed that ‘statistical methods’ are used as just tools here, however, they are vital part of methodology [and so should be given due importance].**

**COMMENTS:** I highly appreciate the study [particularly the objective [Early detection and treatment of adverse events in oncological treatment to improve patients’ safety and outcomes]. However, I have few small {really very ‘minor’} queries. Those are given below. Hope, you will find them worth considering.

1. Although your ‘Article Type: Registered Report Protocol’ it is not indicated anywhere that it is a ‘Protocol’. Preferably the word ‘Protocol’ should appear in title itself. Mention at least in ‘abstract’ is expected.
2. Since ‘Phase I’ is only to assess the feasibility of the application why patients divided into three age groups (<55years, 55-75years, >75years). Do you wish to assess the feasibility of the application for different age groups? You also say that ‘equal-sized’ groups. How was that insured? Use of ‘quota’ sampling may be mentioned.
3. According to ‘abstract-methods’ phase II, will compare the impact of smartphone reported adverse events regarding applied therapy doses and quality of life to those of patients receiving standard care. Here, 36 patients will be randomly assigned to each of the two groups. *Which two groups is not described there*? It is clear only from figure-2 that ‘Patients in group 1: Smartphone questionnaire + standard care’ and Patients in group 2 (control) : Standard care’.
4. In figure-2 Y-axis heading is ‘Total Sample Size’ but generally sample size is given ‘per arm’. I request you to kindly confirm {May please refer to line 124}. Figure-2 is 100% correct, but modified/adjusted/derived.
5. Supporting information files are not named as ‘S1 Table. Smartphone questionnaire for chemotherapies’, ‘S2 Table. Smartphone questionnaire for immunotherapies’ and ‘S3 Table. Feasibility analysis’ as indicated in manuscript. Therefore, ‘S3 Table. Feasibility analysis’ is not available [which is important for me].
6. Refer to section ‘Biometry - sample size estimation’ as well as to lines 148-49 which says regarding Wilcoxon-Mann-Whitney test [Wilcoxon-Mann-Whitney test will be used to assess known-groups validity, as well as 148 to compare differences between NEMO users and patients with standard reporting procedure concerning the severity of occurred adverse events] however, note that ‘Wilcoxon’s Rank Sum’ test and ‘Mann-Whitney U’ test are separate tests [though equivalent]. There is no test by all three together {test called ‘Wilcoxon-Mann-Whitney test’} in my knowledge. Kindly check. (scientist Wilcoxon had given one more test called ‘Wilcoxon’s Signed Ranked test’ which is for paired samples)
7. If feasibility study is already completed [‘S3 Table. Feasibility analysis’ indicates so] then why ‘Phase I’ [which you say is to assess the feasibility of the application] because anyway in this article few sections are only for phase II [though not specified so (which is not desirable, I guess) ex.: section ‘Biometry - sample size estimation’]. Inclusion of ‘Phase I’ may increase confusion.
8. Further comment on (more account but very briefly) on ‘Jonckheere-Terpstra test’ is required, I guess (line 157). Why do you need ‘A distribution-free k-sample test against ordered alternatives’?
